# BrainAgeNeXt: Advancing brain age modeling for individuals with multiple sclerosis

**DOI:** 10.1162/imag_a_00487

**Published:** 2025-02-25

**Authors:** Francesco La Rosa, Jonadab Dos Santos Silva, Emma Dereskewicz, Azzurra Invernizzi, Noa Cahan, Julia Galasso, Nadia Garcia, Robin Graney, Sarah Levy, Gaurav Verma, Priti Balchandani, Daniel S. Reich, Megan Horton, Hayit Greenspan, James Sumowski, Merixtell Bach Cuadra, Erin S. Beck

**Affiliations:** Windreich Department of Artificial Intelligence & Human Health, Icahn School of Medicine at Mount Sinai, New York, NY, United States; Department of Neurology, Icahn School of Medicine at Mount Sinai, New York, NY, United States.; Department of Environmental Medicine and Public Health, Icahn School of Medicine at Mount Sinai, New York, NY, United States.; BioMedical Engineering and Imaging Institute, Icahn School of Medicine at Mount Sinai, New York, NY, United States.; Translational Neuroradiology Section, National Institute of Neurological Disorders and Stroke, National Institutes of Health, Bethesda, MD, United States.; CIBM Center for Biomedical Imaging, Lausanne, Switzerland.; Radiology Department, University of Lausanne and Lausanne University Hospital, Lausanne, Switzerland.

**Keywords:** brain age, aging, multiple sclerosis, MRI, machine learning, deep learning

## Abstract

Aging is associated with structural brain changes, cognitive decline, and neurodegenerative diseases. Brain age, an imaging biomarker sensitive to deviations from healthy aging, offers insights into structural aging variations and is a potential prognostic biomarker in neurodegenerative conditions. This study introduces BrainAgeNeXt, a novel convolutional neural network inspired by the MedNeXt framework, designed to predict brain age from T1-weighted magnetic resonance imaging (MRI) scans. BrainAgeNeXt was trained and validated on 11,574 MRI scans from 33 private and publicly available datasets of healthy volunteers, aged 5 to 95 years, imaged with 3T and 7T MRI. Performance was compared against three state-of-the-art brain age prediction methods. BrainAgeNeXt achieved a mean absolute error (MAE) of 2.78 ± 3.64 years, lower than the compared methods (MAE range 3.55–4.16 years). We also tested all methods across different levels of image quality, and BrainAgeNeXt performed well even with motion artifacts and less common 7T MRI data. In three longitudinal multiple sclerosis (MS) cohorts (273 individuals), brain age was, on average, 4.21 ± 6.51 years greater than chronological age. Longitudinal analysis indicated that brain age increased by 1.15 years per chronological year in individuals with MS (95% CI = [1.05, 1.26]). Moreover, in early MS, individuals with worsening disability had a higher annual increase in brain age compared to those with stable clinical assessments (1.24 vs 0.75,*p*< 0.01). These findings suggest that brain age is a promising prognostic biomarker for MS progression and potentially a valuable endpoint for clinical trials.

## 1 Introduction

The aging population presents a significant global challenge, particularly concerning brain health (Kanasi et al., 2016). As individuals age, the brain undergoes profound structural and functional transformations. These include reductions in brain volume, notably in the prefrontal cortex and hippocampus, and the accumulation of white matter lesions, all of which contribute to a decline in memory, executive function, and overall cognitive capabilities (Peters, 2006). The incidence of neurodegenerative diseases, such as Alzheimer’s disease and Parkinson’s disease, increases with age, further contributing to cognitive deterioration. As the aging population expands, it becomes crucial to deepen our understanding of these neurobiological changes in order to develop effective interventions that can preserve cognitive function and improve the quality of life for the elderly and those affected by neurodegenerative conditions.

Brain age is a biomarker estimated from magnetic resonance imaging (MRI) that detects deviations from healthy aging (Cole & Franke, 2017). The gap between chronological age and estimated brain age is known as brain age difference (BAD). Individuals with a large, positive BAD might be undergoing accelerated aging, while those with a negative BAD might have a healthier brain relative to their chronological age. Brain age can also serve as an imaging biomarker in neurologic and psychiatric diseases. Studies have shown that BAD tends to be higher in people with psychiatric and neurological conditions, including Alzheimer’s disease, multiple sclerosis (MS), and Parkinson’s disease, compared to healthy volunteers (HV) (He et al., 2021;Høgestøl et al., 2019).

MS is an autoimmune disease characterized by inflammatory demyelination and progressive neurodegeneration, leading to physical and cognitive impairment (Reich et al., 2018). Chronological age is strongly associated with the clinical course of the disease (Graves et al., 2023). Individuals who are younger at clinical disease onset are more likely to have a predominantly inflammatory disease course with more total relapses before entering the progressive phase of the disease (Scalfari et al., 2018). This transition from a relapsing-remitting to a progressive clinical course in MS occurs for some but not all individuals. Despite a growing understanding of the disease course and pathophysiological mechanisms, the currently available clinical and biomedical tools are still limited in their ability to identify those at higher risk for progressive disease. With aging, remyelination is less effective, and the restorative efficacy from MS relapses declines alongside reduced plasticity (Conway et al., 2019;Neumann et al., 2019). Older individuals with MS also experience increased rates of brain atrophy (Ghione et al., 2019), which are moderately associated with physical disability and cognitive impairment (Koch, 2021). Currently, MS clinical care relies heavily on MRI findings (Thompson et al., 2018). However, MRI biomarkers, including lesion burden and brain atrophy, have limited utility in predicting cognitive decline and disease progression independent of relapse activity (PIRA). PIRA, often seen in people with MS (pwMS), is likely driven, in part, by neurodegeneration (Sumowski et al., 2018). There is currently a lack of effective outcome measures for proof-of-concept clinical trials in progressive MS, where disability accumulation is too slow to be a feasible assessment metric (van Munster & Uitdehaag, 2017).

While chronological age is an important factor in the disease course, brain age may provide additional insights into disease progression by capturing subtle neurodegenerative changes. Studies suggest that accelerated brain aging may offer unique prognostic value (Elliott et al., 2021). For instance, individuals with a “healthy” brain at baseline, characterized by minimal atrophy and lesion burden, may exhibit a slower progression of physical and cognitive disability (Popescu et al., 2013;Sumowski et al., 2016). Incorporating brain age alongside traditional MRI biomarkers could improve predictions of cognitive decline and progressive disease, addressing a critical gap in clinical care for progressive MS.

Most brain age prediction models applied to MS have traditionally relied on classical machine-learning methods, which are trained using previously extracted brain volumetric features (Cole et al., 2020;Denissen et al., 2021;Høgestøl et al., 2019). This approach typically involves segmenting the brain into various structures and then extracting volumetric or morphological features from these regions using brain imaging software such as FreeSurfer (Fischl, 2012) or FSL (Jenkinson et al., 2012). However, brain parcellation tools often yield inconsistent results when applied to MRI scans obtained from different sites or using different imaging parameters (Guo et al., 2018;S. Liu et al., 2020). Thus, relying on pre-extracted brain volumetric features can limit the applicability of these models to real-world clinical settings, where scans from various sources and conditions are common, or to research studies that include multi-site data. Additionally, while volumetric features provide useful information, they may only partially represent the intricate patterns of brain aging across the human lifespan. For example, subtle changes in cortical microstructure, alterations in white matter integrity, and surface curvature properties (Dos Santos Silva et al., 2023) are critical aspects of aging that may be missed by traditional segmentation-based tools. Additionally, MRI data inherently contain rich metabolic and microstructural tissue-level information that extends beyond simple volumetric measurements. For instance, subtle intensity-based texture patterns, local microstructural signatures, and signal heterogeneity—all influenced by factors such as myelination (Callaghan et al., 2014), iron deposition (Bilgic et al., 2012), and axonal integrity (Storsve et al., 2016)—are known to evolve with age and can precede overt volumetric atrophy (Raz et al., 2007). These limitations underscore the importance of transitioning to deep learning models capable of leveraging raw MRI data to capture the full spectrum of brain aging patterns, which have recently started to be used to assess brain age (Brier et al., 2023).

In recent years, convolutional neural networks (CNNs) have demonstrated exceptional performance in image analysis tasks, including medical imaging. These networks can automatically learn and extract relevant features from high-dimensional data, making them competitive for complex tasks such as brain age prediction. CNNs provide a more flexible and robust alternative by directly learning from raw and heterogenous MRI data without relying on several preprocessing steps or predefined features (Bashyam et al., 2020;La Rosa et al., 2022;Lee et al., 2022;Wood et al., 2022). Recent state-of-the-art brain age models have employed advanced deep learning techniques, achieving high accuracy and robustness in their predictions (Leonardsen et al., 2022). These models leverage large datasets and architectures such as CNNs and graph neural networks to capture detailed patterns in MRI data (Lee et al., 2022;Moon et al., 2024), significantly outperforming traditional machine-learning methods (Dörfel et al., 2023). While CNNs are now widely used for brain age analysis, their reliance on local receptive fields and fixed-size convolutional kernels may limit their ability to capture long-range dependencies in brain MRI data. Transformers, with their self-attention mechanisms, offer a promising path for improving robustness and generalizability in brain age prediction.

In this study, we propose a novel CNN, based on the MedNeXt architecture and inspired by transformer designs, for the brain age estimation task. MedNeXt integrates transformer-inspired mechanisms such as hierarchical feature learning and efficient global context modeling within a convolutional framework to capture both local and global patterns. By utilizing the advanced capabilities of CNNs, we aim to overcome the limitations associated with classical machine-learning approaches and improve the robustness and accuracy of brain age estimation in MS. Brain age estimation has mostly been explored at 1.5T and 3T (Dörfel et al., 2023;Dufumier et al., 2022). While 7T MRI has led to important insights into neurologic diseases, including MS (Filippi et al., 2014;Ineichen et al., 2021), only one study to date has explored brain age measurement using 7T MRI (Verma et al., 2022). Verma et al. proposed a regression-based model for predicting brain age from 7T MRI volumetric features quantified using FreeSurfer (Fischl, 2012). In our study, we include 7T MRI data in the training and testing datasets of our deep learning-based model, leveraging its enhanced resolution and tissue contrast to potentially improve the brain age prediction accuracy.

In this work, we analyze a substantial dataset of over 11,000 1.5T, 3T, and 7T MRI scans, spanning most of the human lifespan, to characterize typical aging patterns within the healthy population. Our contribution is twofold: first, we propose BrainAgeNeXt, a novel CNN architecture inspired by the MedNeXt framework, for estimating brain age from diverse T1-weighted (T1w) MRI scans. We evaluate BrainAgeNeXt on large, multi-site cohorts of HV imaged with both 3T and 7T MRI. Second, we extend the application of the brain age paradigm to three longitudinal cohorts of subjects with MS to characterize brain age in MS.

## 2 Methods

### 2.1 Datasets

The datasets used in this study were anonymized, and as a result, the work is classified as non-human research.

### 2.2 Healthy subjects

**Fig. 1.**
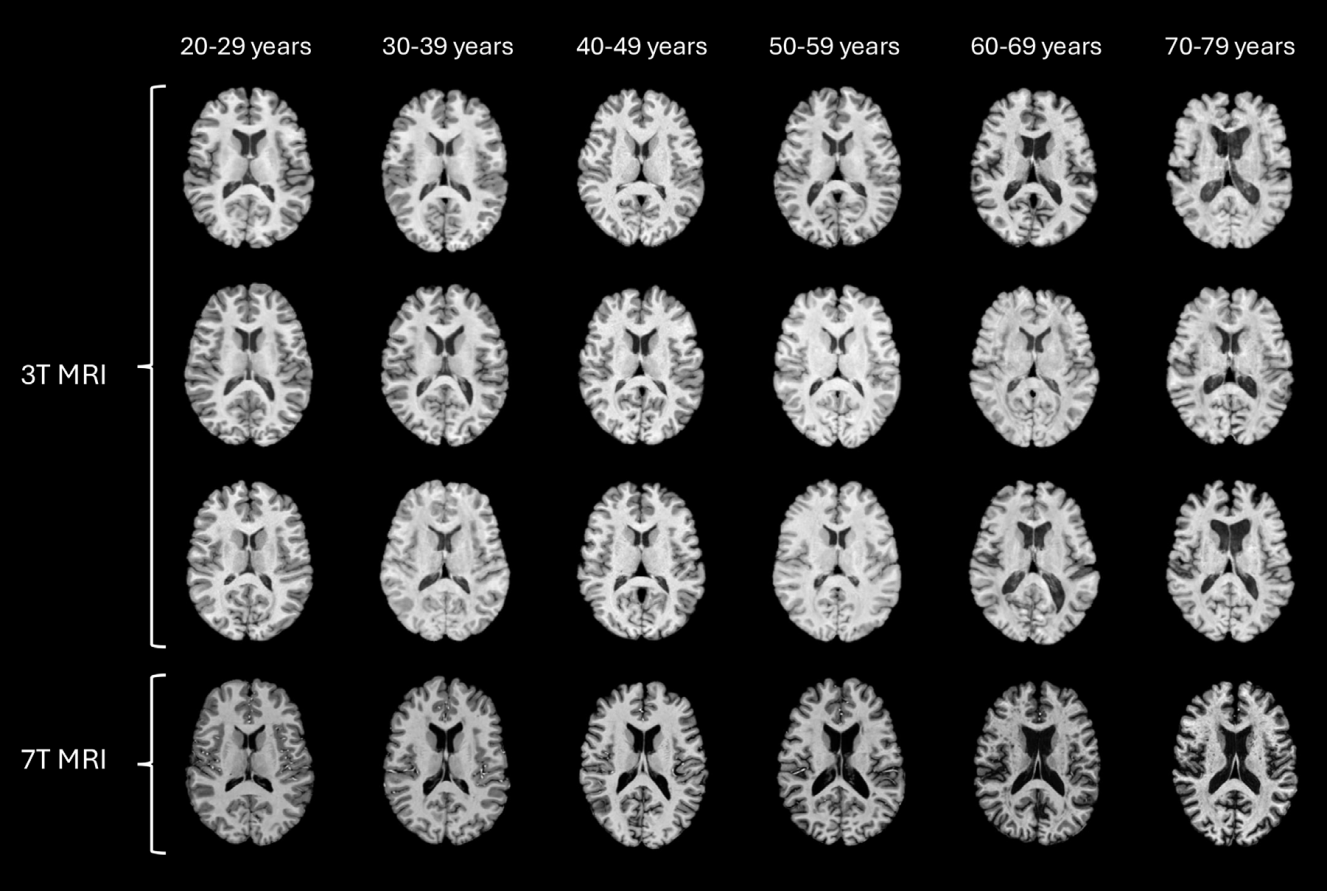
Representative axial MRI slices from healthy subjects across different decades. The image illustrates age-related anatomical changes. Different T1w sequences are shown for the 3T images, whereas the 7T scans are all MP2RAGE.

**Fig. 2.**
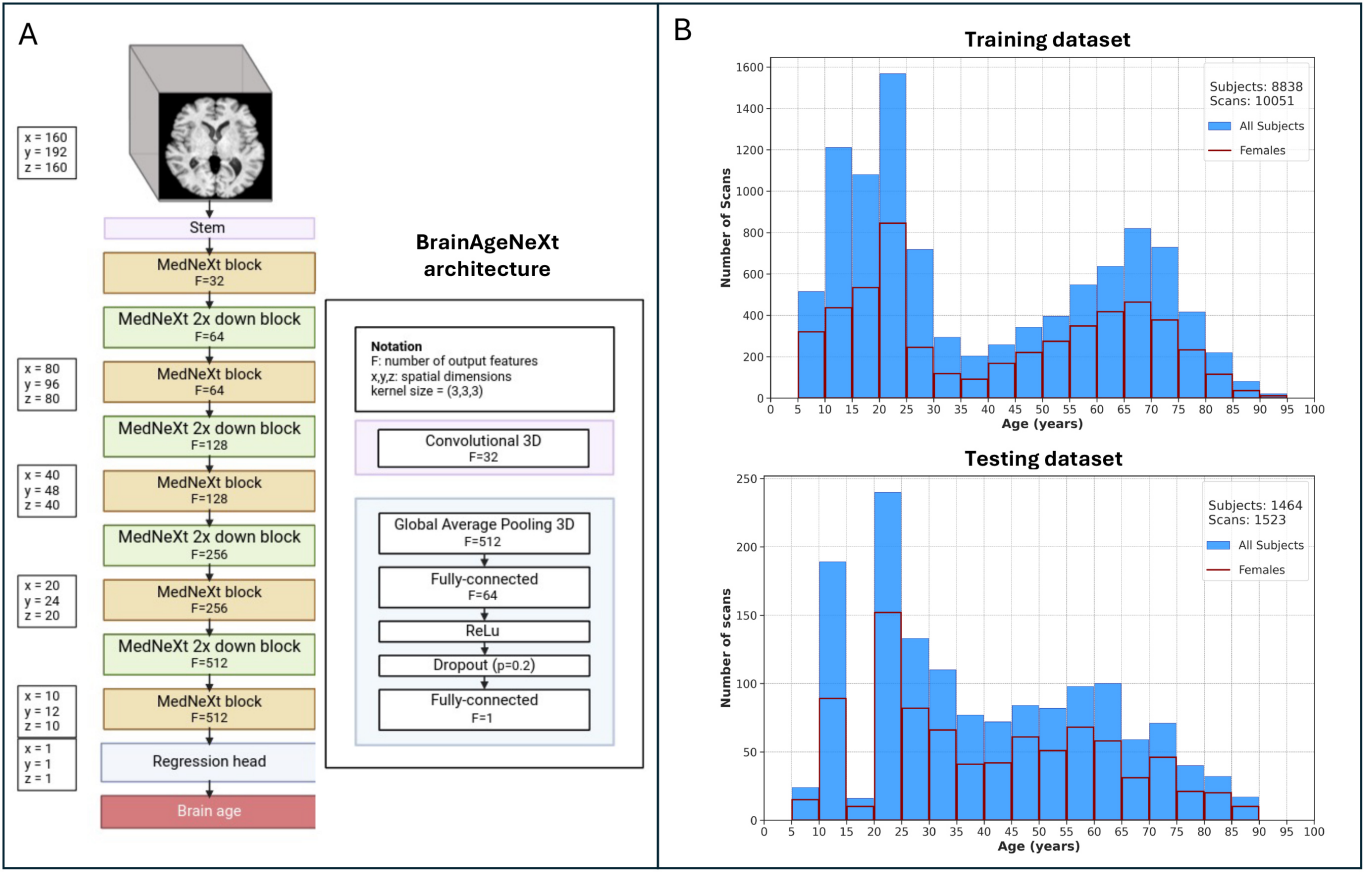
BrainAgeNeXt architecture and training/testing datasets. (A) Scheme of the proposed BrainAgeNeXt architecture. (B) Histograms showing the age distribution of the HV training and testing MRI scans.

We aggregated several publicly available and private datasets of HV imaged at 1.5T, 3T, and 7T, to obtain a dataset of 11,574 T1w MRI (Fig. 1). The HV MRI scans used for training and validation (*n*= 10,051, from 8,838 unique subjects) are shown inTable 1. The age range is 5–95 years (mean 39.2 ± 24.0 years), and 51% are female. The training set included MRI images acquired at 75 sites with various scanners and imaging protocols. Various T1w MRI sequences were included: spoiled gradient-recalled echo (SPGR), magnetization-prepared rapid gradient echo (MPRAGE), and magnetization-prepared two rapid acquisition gradient echoes (MP2RAGE) (Marques et al., 2010). For testing, we selected 1,523 MRI scans from 10 sites, unseen during training, with 3T or 7T MRI acquired with Siemens, Philips, or GE scanners. Some datasets contained two scans of a single subject, either at different time points (n = 50) or magnetic field strengths (n = 9). The age range of the testing set is 8–89 years (mean 39.1 ± 20.9 years), and 59% are female (Table 2,Fig. 2B).

**Table 1.** Datasets used for training BrainAgeNeXt.

Training datasets	Field strength	T1w sequence	Number of subjects (age range)	Sex (F)
openBHB ( Dufumier et al., 2022 )	1.5T, 3T	T1w, SPGR, MPRAGE	5,330 (5-86y)	54%
Cam-CAN ( Taylor et al., 2017 )	3T	MPRAGE	653 (18-87y)	49%
Oasis 3 ( LaMontagne et al., 2019 )	3T	MPRAGE	755 (42-95y)	48%
Oasis 4 ( Koenig et al., 2020 )	3T	MPRAGE	663 (21-94y)	54%
QTAB ( Strike et al., 2023 )	3T	MPRAGE	510 (9-16y)	52%
NKI-RS ( Nooner et al., 2012 )	3T	MPRAGE	653 (6-85y)	51%
PREVENT-AD ( Tremblay-Mercier et al., 2021 )	3T	MP2RAGE	43 (57-82y)	51%
ATAG (Forstmann et al., 2014 )	7T	MP2RAGE	53 (19-75y)	64%
CEREBRUM-7T ( Svanera et al., 2021 )	7T	MP2RAGE	76 (19-40y)	48%
CoRR ( Zuo et al., 2014 )	7T	MP2RAGE	22 (22-30y)	45%
CFFM ( Haast et al., 2020 )	7T	MP2RAGE	32 (20-66y)	47%
Mount Sinai ( Brown et al., 2019 )	7T	MP2RAGE	48 (20-70y)	55%

MPRAGE: Magnetization Prepared Rapid Gradient Echo; MP2RAGE: Magnetization Prepared 2 Rapid Gradient Echoes; SPGR: Spoiled Gradient Recalled Echo.

### 2.3 Subjects with multiple sclerosis

Three longitudinal cohorts of pwMS were included in this study:

- Reserve Against Disability in Early Multiple Sclerosis (RADIEMS, Mount Sinai) (Brandstadter et al., 2019): a longitudinal study investigating factors associated with disability progression in pwMS diagnosed within 5 years at the time of enrollment. One hundred and eighty-five pwMS (165 relapsing remitting MS (RRMS), 20 clinically isolated syndrome; disease duration 2.2 ± 1.4 years; mean age at enrollment 34 years (range 20–50); 67% female) underwent 3T MRI and clinical assessment, which included extensive cognitive testing at baseline (2016–2018) and at year 3 follow-up visits.
- Longitudinal Cortical Lesion study (LCL, National Institutes of Health [NIH]) (Beck et al., 2024): 64 pwMS (45 RRMS, 19 progressive MS; disease duration 14.2 ± 10.9 years; mean age 46 years (range 20–77); 64% female) underwent 3T and 7T MRI and clinical assessment at baseline (2017–2018), year 1 and year 2. At baseline, the median interval between 3 and 7 T MRI was 9 weeks (range 1–34).
- Longitudinal 7T (Long 7T, Mount Sinai): longitudinal study of advanced lesional biomarkers in newly diagnosed pwMS with 7T MRI and clinical assessments (2022-present). This includes 28 people with RRMS (disease duration 0.8 ± 0.4 years, mean age 34 years (range 24–54), 62% female).

**Table 2.** Testing datasets.

Dataset	Field strength	Scanner	T1w sequence ^a^	Number of subjects (age range)	Sex (% female)
MR-ART ( Nárai et al., 2022 )	3T	Siemens Prisma	MPRAGE	148 (18–75y)	64%
DLBS ( Kennedy et al., 2015 )	3T	Philips	MPRAGE	315 (10-81y)	63%
SALD ( Wei et al., 2018 )	3T	Siemens Tim Trio	MPRAGE	493 (19-80y)	62%
Horta et al. (2023)	3T	Philips Achieve	MPRAGE	87 (18-81y)	51%
PROGRESS	3T	Philips whole-body system	MPRAGE	215 (8-15y)	49%
RADIEMS ( Brandstadter et al., 2019 )	3T	Siemens Skyra	MPRAGE	32 (24-49y)	64%
NIMH ( Nugent et al., 2022 )	3T	GE Discovery MR750	SPGR	50 (21-67y)	47%
NIMH ( Nugent et al., 2022 )	3T	GE Discovery MR750	MPRAGE	68 (21-71y)	46%
S. Liu et al. (2024)	3T	Siemens Prisma	MPRAGE	44 (19-45y)	66%
Chen et al. (2023)	3T	Siemens Prisma	MPRAGE	10 (25-41y)	30%
Chen et al. (2023)	7T	Siemens Terra	MP2RAGE	9 (25-41y)	33%
CBS ( Tardif et al., 2016 )	7T	Siemens Magnetom	MP2RAGE	28 (21-36y)	54%
NIH ( Beck et al., 2022 )	7T	Siemens Magnetom	MP2RAGE	5 (40-78y)	60%
Mount Sinai ( Silva et al., 2023 )	7T	Siemens Magnetom	MP2RAGE	19 (25-76y)	55%

a 3T images were acquired at a 1.0-1.5 mm^3^resolution, 7T images had a 0.5-0.8 mm^3^resolution.

MPRAGE: Magnetization Prepared Rapid Gradient Echo; MP2RAGE: Magnetization Prepared 2 Rapid Gradient Echoes; SPGR: Spoiled Gradient Recalled Echo.

For all cohorts, the clinical assessment included Expanded Disability Status Scale (EDSS), Symbol Digit Modalities Test (SDMT), Nine-Hole Peg Test (9HPT), and Timed 25-Foot Walk (T25FW). For the LCL cohort, the written version of the SDMT was performed, whereas the oral version was used for the RADIEMS and Long 7T cohorts.

### 2.4 Preprocessing

All T1w MRI scans underwent skull-stripping with SynthStrip, including CSF removal (Billot et al., 2023), N4 bias field correction with default parameters, and linear registration with 6 degrees of freedom to the MNI152 1 mm isotropic space with ANTs (Avants et al., 2011). We then applied Z-score normalization to all MRI images, considering only non-zero voxels within the brain to ensure that the background did not influence the normalization process.

To determine the relationship between brain volumetric measures and brain age predictions, tissue segmentation was performed on all HV testing images using SynthSeg (Freesurfer 7.4.1 (Billot et al., 2023;Fischl, 2012)). For RADIEMS, MS lesions were segmented semiautomatically (La Rosa et al., 2020) using FLAIR images. For LCL, MS lesions were segmented semiautomatically using 3T FLAIR and T1w images (Beck et al., 2024). For all 3T scans of pwMS, lesion filling was performed using FSL (Jenkinson et al., 2012) within the white matter (WM) tissue mask from SynthSeg, followed by manual correction.

### 2.5 Network architecture

We propose BrainAgeNeXt, a CNN inspired by the MedNeXt framework (Roy et al., 2023), which predicts brain age from minimally preprocessed 3D T1w MRI. BrainAgeNeXt architecture consists of the MedNeXt encoder, composed of four MedNeXt blocks, followed by a regression header. The MedNeXt blocks are a 3D extension of the ConvNeXt networks (Z. Liu et al., 2022), which incorporate architectural insights derived from Vision Transformers and Swin Transformers (Z. Liu et al., 2021) into a convolutional framework. Each MedNeXt block consists of three layers that mirror transformers’ principles. The first layer, a depthwise convolution, captures spatial features independently for each channel, while the second layer, an expansion layer, enhances feature representation with additional channels. Finally, a compression layer uses a pointwise convolution to perform a channel-wise compression of the feature maps. This design mimics self-attention mechanisms, balancing computational efficiency and expressive power. Further details are available in the original MedNeXt paper (Roy et al., 2023).

Following the fourth MedNeXt block, the regression head first applies 3D global average pooling to spatially aggregate feature maps into a compact representation, reducing dimensionality while preserving critical global information. Finally, two fully connected layers are added to provide a unique value, brain age, as output. These last two layers use an ReLU activation function, and the first one has a dropout rate of 0.2 to mitigate overfitting.

### 2.6 Data augmentation

Several intensity and spatial transformations were applied to the input data during training to improve the generalizability of BrainAgeNeXt. The spatial transformations consisted of random rotations along the three axes (up to 0.1 radiant), affine transformations, zooming in and out (up to 5% of the image size), and foreground cropping and padding to the input dimensions (160,192,160). The intensity augmentation included random contrast adjustment, gamma transformations, simulated bias field, random noise, and random swapping patches within the image. Moreover, random motion and ghosting artifacts were simulated (Pérez-García et al., 2021). All transforms were applied with a random 0.2 probability at each training iteration. MONAI (Consortium, 2020) and TorchIO (Pérez-García et al., 2021) python libraries were used for these transformations.

### 2.7 Configuration

The network was trained for 200 epochs using the L2 loss function, with a batch size set to 4. The code is a custom Python script based on the MONAI framework (Consortium, 2020). Training was performed on one NVIDIA A100 80 GB GPU and took approximately 6 days. From the training set (Table 1), we selected an internal and an external validation dataset. The internal validation set consisted of 5% of the training set, randomly sampled but balanced to ensure representation across each 5-year interval throughout the lifespan. The external validation dataset included MRI scans from an unseen site during training (n = 498), covering an age range of 6–84 years. The internal and external validation datasets were used exclusively for model evaluation and were not included in the training process. We saved the best model based on the lowest average of the internal and external validation losses, ensuring robust performance across diverse datasets. The testing set consists of MRI scans (n = 1,523) acquired at 10 sites unseen during training. Our code is made publicly available^1^.

### 2.8 Brain age prediction

The output provided by the network is corrected for the known regression to the mean bias, which causes brain age overestimation for younger subjects and underestimation for older ones (Cole & Franke, 2017). To achieve this, we applied the correction proposed byBeheshti et al. (2019):

BAD=α*chronological age+β 

whereαandβare obtained by fitting a linear regression model into the validation dataset. The final brain age prediction is derived from an ensemble of five independently trained models. These models share identical configurations but have random weight initializations, and the median of their outputs is used as the final brain age prediction of BrainAgeNeXt.

### 2.9 Baselines

The DenseNet convolutional neural network architecture (Huang et al., 2018), along with its 3D extension, have been used for the brain age estimation task in the literature (Dörfel et al., 2023;Wood et al., 2022). We trained one DenseNet architecture as a baseline model to compare with our proposed BrainAgeNeXt using the same training dataset. BrainAgeNeXt, inspired by the ConvNeXt (Z. Liu et al., 2022) and MedNeXt (Roy et al., 2023) architectures, differs from the baseline DenseNet by incorporating advanced convolutional techniques for improved feature extraction, performance, and generalization in brain age prediction. In addition, we tested two publicly available methods, one CNN and one regression-based model, that in a recent review resulted as the top performing brain age estimation methods (Dörfel et al., 2023). All three methods were tested on the same testing datasets as BrainAgeNeXt.

- **DenseNet**:Wood et al. (2022,^2024^) proposed to train a DenseNet201 to estimate brain age from routine clinical MRI data. When trained on over 19,000 MRI scans (18–96 years), the model achieved a mean age error (MAE) < 4.0 years, in line with other state-of-the-art approaches (Bashyam et al., 2020;Cole & Franke, 2017;Franke & Gaser, 2019). We trained this architecture using our training dataset (mean age 39.2 ± 24.0 years, range 5–95, 51% female) using a random weight initialization and the same data augmentation and validation strategy as for BrainAgeNeXt.
- **pyment (sfcn-reg)**:Leonardsen et al. (2022)developed a shallow 3D convolutional neural network trained from skull-stripped T1w MRI registered to the MNI152 space. FreeSurfer was used for skull-stripping and FSL*FLIRT*for linear registration. The network was trained with 34,285 HV from 21 datasets (age range 3–95 years, 52% female). For our evaluation, we applied the publicly available trained model provided.
- **brainageR**: A brain age estimation method based on Gaussian process regression, a nonparametric Bayesian regression approach (Clausen et al., 2022;Cole et al., 2018). Raw T1w MRI scans were segmented with SPM12 into gray matter, white matter, and CSF (Schmidt et al., 2012). Their probability maps were normalized and concatenated. Following a principal component analysis, a Gaussian progress regression model was trained to predict brain age. This method was previously trained on 2,277 HV (age 40.6 ± 21.4 years, range 18–92, 48% female). As some of these HV were included in our testing dataset, we evaluated brainageR only on the remaining scans (*n*= 352, mean age 35.3 ± 19.2 years, range 18–80, 48% female). Subjects younger than 18 years were excluded from this analysis as brainageR was trained only with subjects 18 and older.

### 2.10 Statistical analysis

To estimate the accuracy of brain age prediction in HV, we computed the mean absolute error (MAE):

$$MAE=\frac{1}{N}\;\sum_{i=1}^{N}\left|{\hat{y}}_{i}-y_{i}\right|$$

where N is the number of subjects,${\hat{y}}_{i}$is the brain age prediction for subject i, and$y_{i}$is their chronological age.

The MAE represents the average absolute difference between the predicted brain age and the chronological age. For healthy subjects, the MAE should be close to zero. Pearson’s*r*was calculated to assess the correlations between predicted brain age and chronological age, as well as between predicted brain age and BAD. Ideally, for an unbiased model, Pearson’s*r*for the correlation between chronological age and brain age should be 1, while correlation between chronological age and BAD should be 0. Using the Mann-Whitney U test, we assessed whether the differences in the MAE between different brain age models are statistically significant, while also considering the effect size to understand the practical significance of these differences. Confidence intervals were calculated using the bootstrap method with 1,000 resamples, ensuring robust and non-parametric estimation of variability.

Monotonic correlations between MRI markers or clinical measures and BAD were evaluated using Spearman’s rank correlation. Statistical significance was determined using a*p*-value threshold of 0.05, adjusted for multiple comparisons using the Benjamini-Hochberg false discovery rate (FDR) correction.

## 3 Results

### 3.1 Healthy volunteers

The results of the HV analysis are summarized inTable 3andFigure 3. In the large testing dataset, consisting of 1,523 HV MRI scans, our proposed BrainAgeNeXt achieves the lowest MAE of 2.78 ± 3.64 years, followed by DenseNet (MAE = 3.55 ± 3.90 years), pyment (MAE = 3.59 ± 3.66 years), and brainageR (MAE = 4.46 ± 5.14 years). No statistically significant difference in BAD between males and females was observed for BrainAgeNeXt (*p*= 0.37). Considering only subjects not included in brainageR training set who are 18 years old or older (n = 352), BrainAgeNeXt still outperformed all other approaches (Supplementary Table 1). Analyzing 7T MRI scans separately (n = 61), the MAE for BrainAgeNeXt is 2.08, versus 4.12 for DenseNet. DenseNet had a U statistic of 1,929 (*p*= 0.73) compared to BrainAgeNeXt, with a rank-biserial correlation r = 0.52, indicating a large yet non-significant effect size. For pyment, the U statistic was 938 (*p*< 0.001) compared to BrainAgeNeXt, with r = 0.25, indicating a small effect size. Additionally, all models were compared on the MR-ART dataset, which presents three T1w scans for each of its 148 HV: one without motion artifacts, one with moderate motion artifacts, and one with stronger motion artifacts (Table 3). While BrainAgeNeXt outperforms the other methods across all three sets of images, its MAE increases significantly between artifact free images and those with strong motion artifacts (*p*= 0.03).

**Table 3.** Performance of BrainAgeNeXt vs state-of-the-art models.

				Testing dataset ( *n* = 1,523)			7T MRI ( *n* = 61)		
Model				MAE (years)	BA vs CA (Pearson’s *r* )	BAD vs CA (Pearson’s *r* )	MAE (years)	BA vs CA (Pearson’s *r* )	BAD vs CA (Pearson’s *r* )
BrainAgeNeXt				**2.78**	0.985***	**-0.010**	**2.08**	0.982***	**0.001**
DenseNet				3.55	0.978***	-0.212**	4.12	0.967***	-0.337
Pyment				3.59	0.972***	-0.309***	7.98	0.683***	0.655***
BrainageR				4.46	0.965***	-0.202**	12.22	0.318***	0.736***

MR-ART ( *n* = 148)									
	Motionless			Moderate motion			Strong motion		
Model	MAE (years)	BA vs CA (Pearson’s *r* )	BAD vs CA (Pearson’s *r* )	MAE (years)	BA vs CA (Pearson’s *r* )	BAD vs CA (Pearson’s *r* )	MAE (years)	BA vs CA (Pearson’s *r* )	BAD vs CA (Pearson’s *r* )
BrainAgeNeXt	**2.82**	0.968***	**-0.043**	**3.16**	0.947***	**-0.022**	**3.50**	0.942***	0.062
DenseNet	3.26	0.952***	0.061	3.49	0.945***	0.163*	3.98	0.932***	**0.055**
Pyment	3.18	0.947***	-0.382***	3.39	0.939***	-0.155**	3.92	0.945***	-0.187**
BrainageR	3.17	0.970***	-0.230**	5.48	0.830***	-0.325***	7.41	0.811***	-0.336***

Bold font indicates the best performance for each metric across models.

MAE: mean age error; BA: brain age; CA: chronological age; BAD: brain age difference.

Significance levels: *p< 0.05, **p< 0.01, ***p< 0.001.

**Fig. 3.**
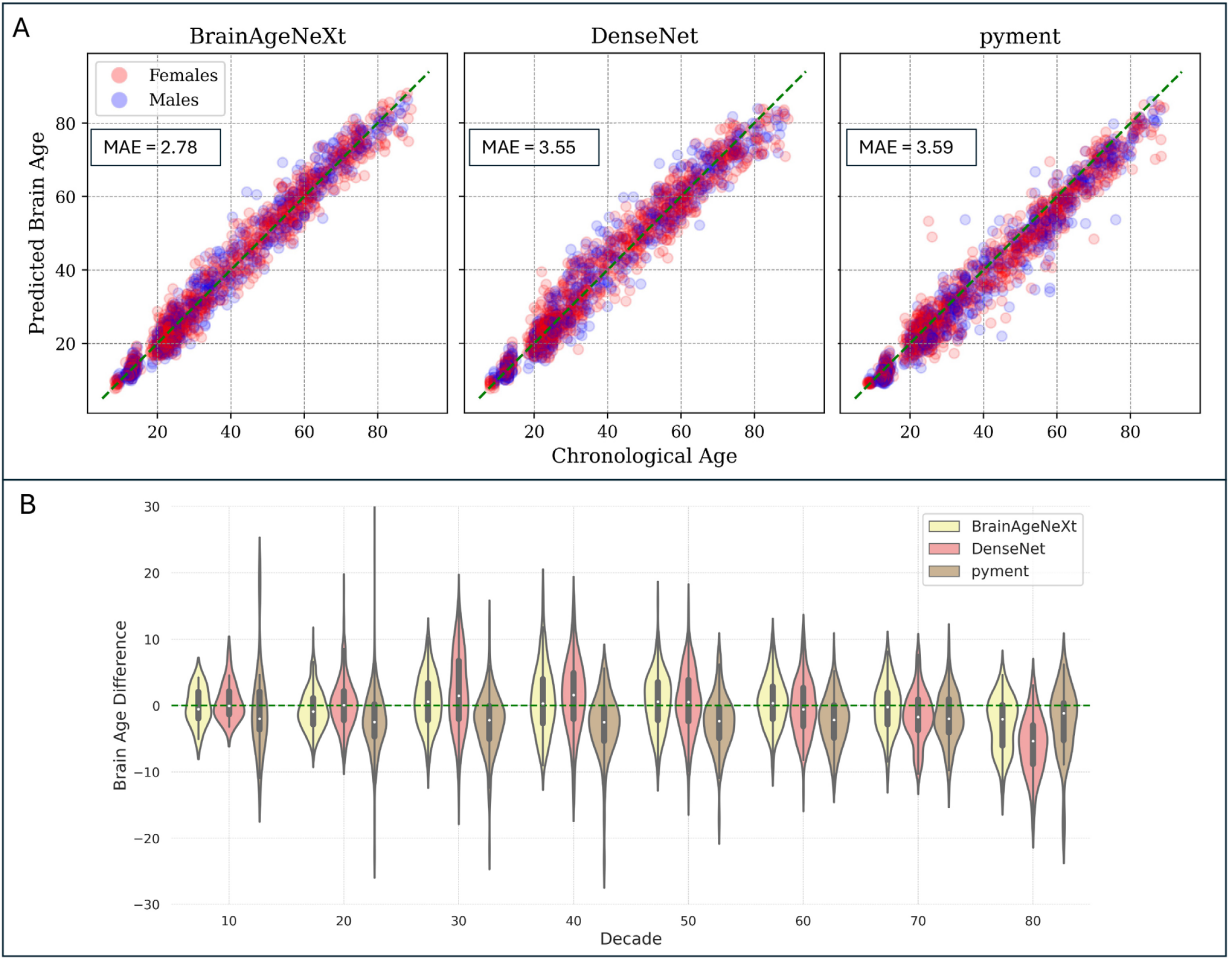
BrainAgeNeXt accurately predicts brain age in healthy volunteers. (A) Scatter plots comparing predicted brain age versus chronological age for BrainAgeNeXt, DenseNet, and pyment in the testing dataset (n = 1,523). (B) Violin plot showing the BAD for the three methods across all decades. Performance of BrainageR is not depicted as it was only tested on a subset of the overall testing set.

The variance between the testing set predictions of the five BrainAgeNeXt models had a mean of 2.61 ± 2.38 years, with a range of 0.02 to 19.37, indicating moderate agreement across models for most samples, although certain subjects had a greater variability. The testing set’s MAE for the five BrainAgeNeXt models ranged from 2.72 to 2.94 years, showing consistent performance across the ensemble models.

**Fig. 4.**
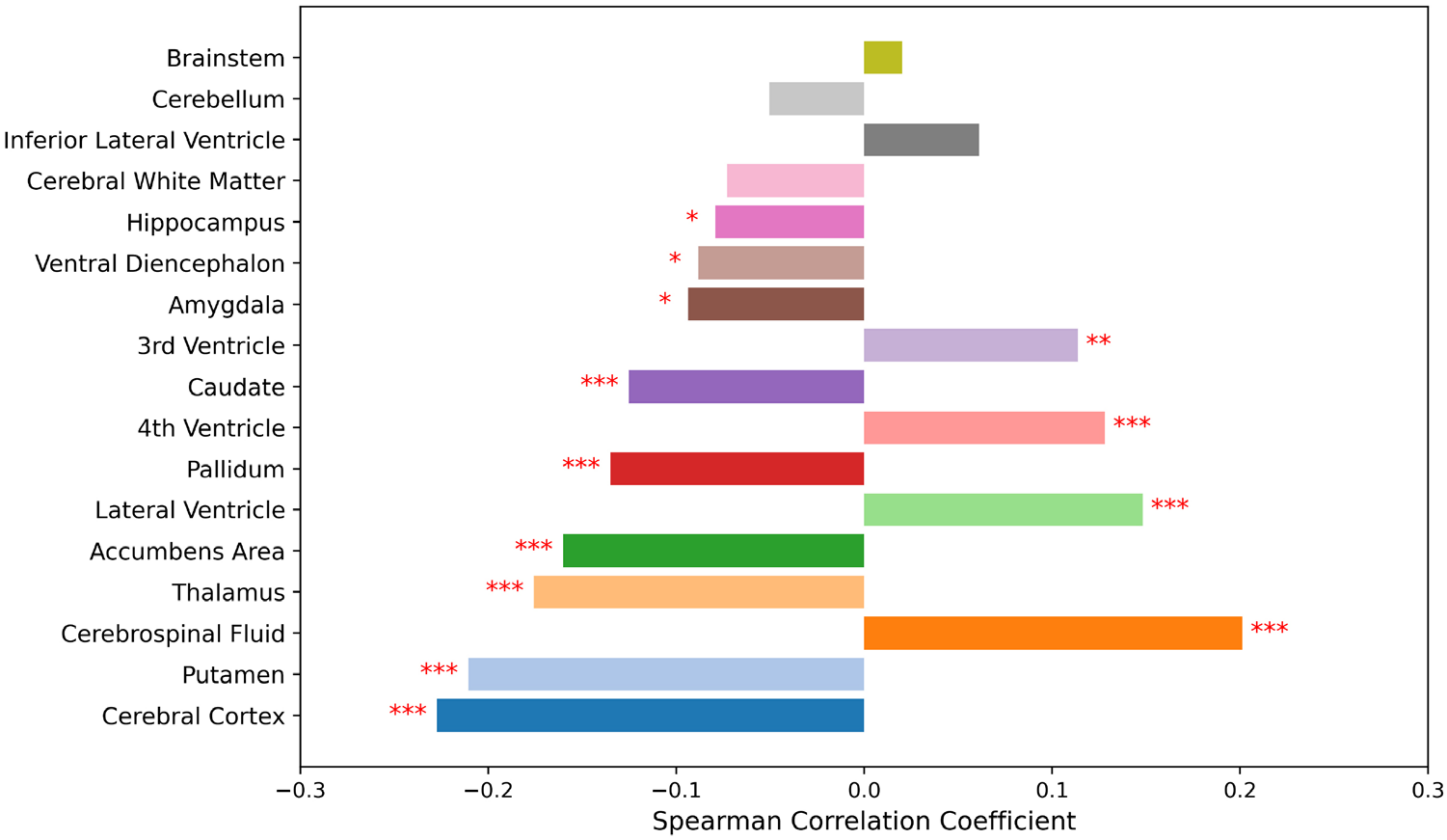
BrainAgeNeXt predictions are strongly correlated with gray matter and cerebrospinal fluid volumes. Correlations between brain parcels (normalized for intracranial brain volume) and BAD. Brain parcellation was performed with SynthSeg (FreeSurfer) (Billot et al., 2023). Significance level: *p< 0.05, **p< 0.01, ***p< 0.001.

To better understand BrainAgeNeXt predictions, we investigated the correlations between brain parcel volumes as obtained with FreeSurfer (Billot et al., 2023) and BAD in the testing dataset for all subjects aged 21 and older (Fig. 4,Supplementary Fig. 1). We combined parcels from the left and right hemispheres, and each parcel volume was normalized by the total intracranial volume. Correlations with BAD were low to moderate, with the strongest correlations for the cerebral cortex (*r*= -0.23,*p*< 0.001), putamen (*r*= -0.21,*p*< 0.001), and cerebrospinal fluid (*r*= 0.20,*p*< 0.001).

### 3.2 BrainAgeNeXt brain age predictions in people with MS (pwMS)

We predicted brain age on three longitudinal cohorts of pwMS using our proposed BrainAgeNeXt.

#### 3.2.1 Cross-sectional analysis

**Fig. 5.**
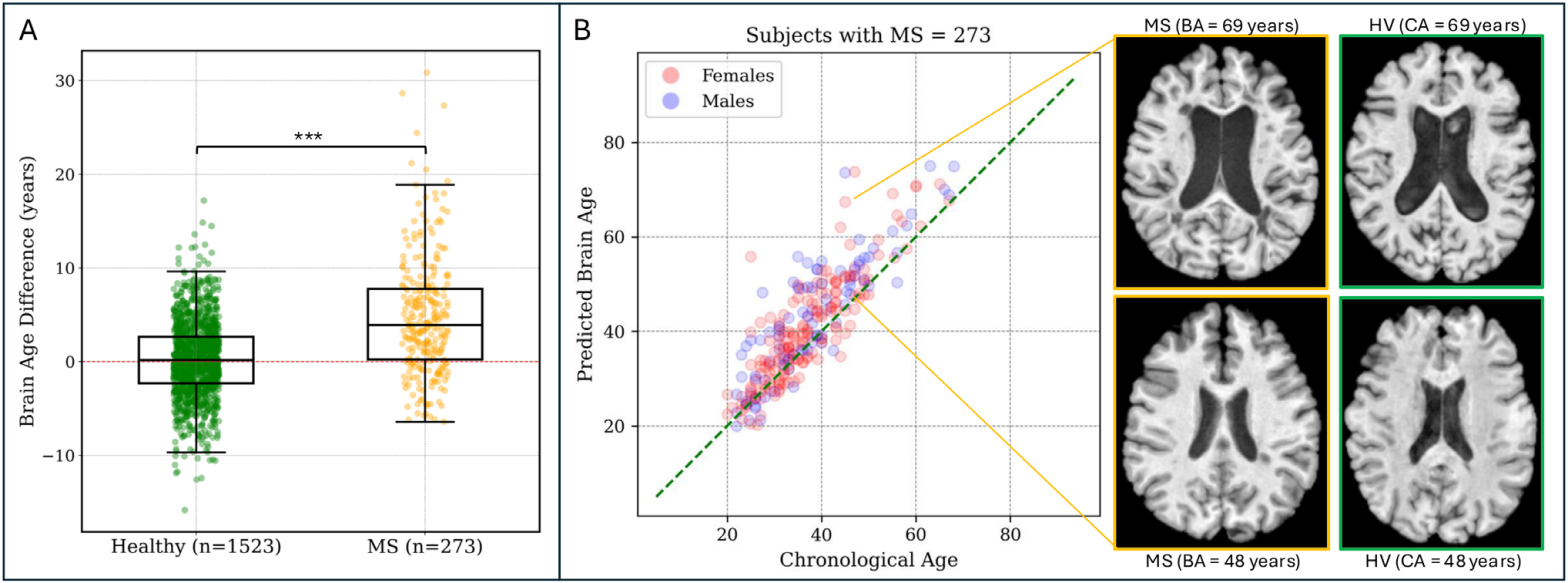
Brain age difference is greater in individuals with MS compared to HV. (A) The predicted brain age for pwMS is on average 4.20 years greater than their chronological age. Significance level: ***p< 0.001. (B) Correlation between predicted brain age and chronological age in individuals with MS. A representative axial MRI slice of two 45-year-old individuals with MS is shown on the right. The individual with a brain age of 69 years has a greater degree of brain parenchymal atrophy and ventricular enlargement.

We considered all pwMS from the LCL (3T MRI scans), RADIEMS, and Long 7T cohorts. pwMS had a higher median BAD of 4.21 years (range: -7 to 29, IQR: 8.23) compared to HV in the testing dataset, who had a mean BAD of 0.02 years (range: -13 to 17, IQR: 4.41),*p*< 0.001 (Fig. 5). No significant difference was observed in predicted brain age between males and females (*p*= 0.09). However, the trend indicated slightly worse performance for men.

**Fig. 6.**
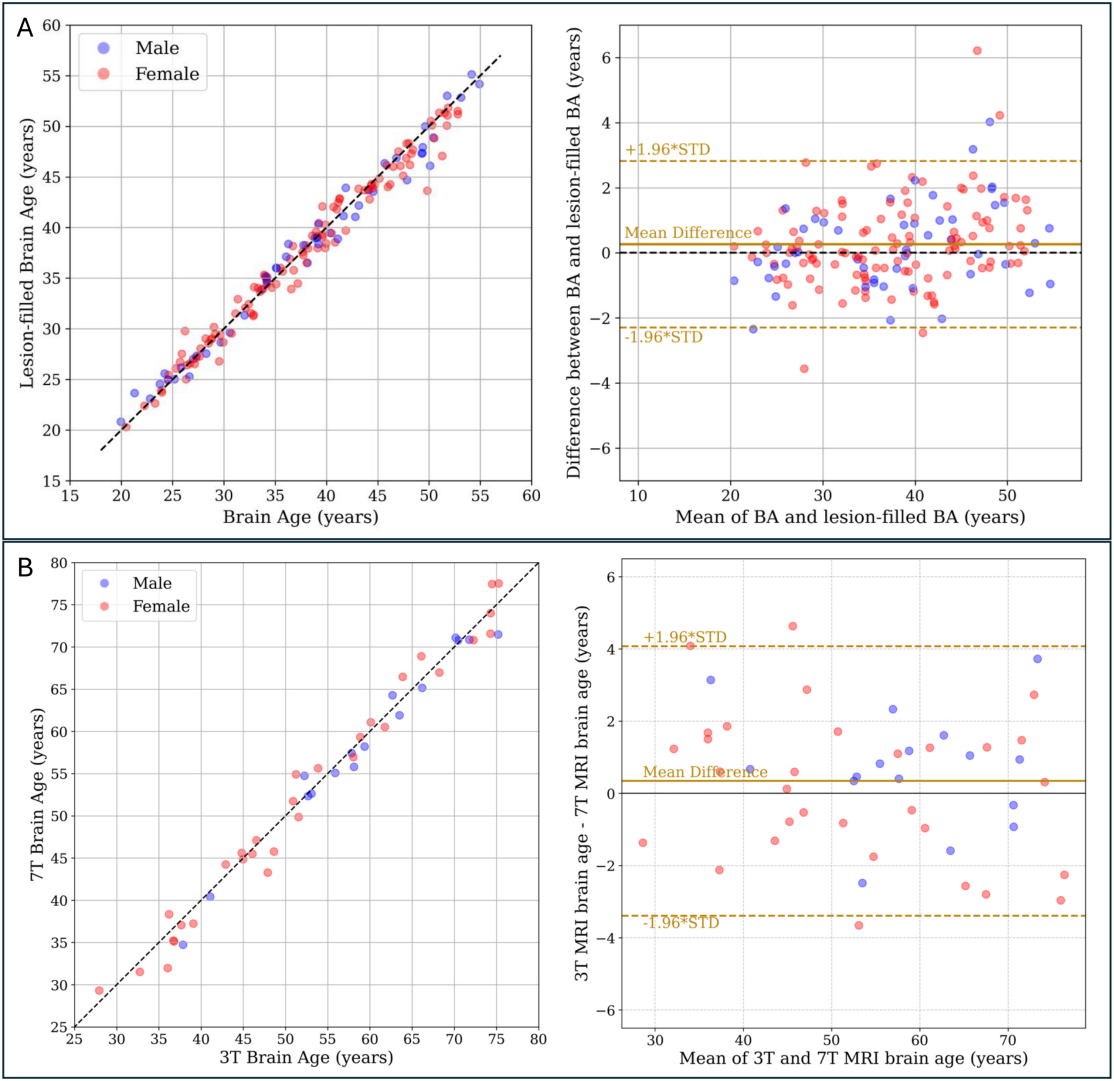
Predicted brain age is similar in lesion-filled and non-lesion-filled images and between 3T and 7T images from the same subjects. (A) Comparison of the predicted brain age from T1w MRI before and after lesion filling in people with multiple sclerosis (pwMS) from the RADIEMS cohort (*n*= 162). The two values are strongly correlated (Spearman’s ρ = 0.99,*p*< 0.001), with a mean difference of 0.26 years (right upper plot, solid golden line). (B) A comparison of the brain age predictions from 3T and 7T MRI for pwMS in the LCL cohort (n = 47). There is a strong correlation between brain age from 3T and 7T images (Spearman’s ρ = 0.99,*p*< 0.001). BA: brain age.

We explored the effect of MS lesions on the predicted brain age. For a subset of pwMS from the RADIEMS cohort (*n*= 162), we tested BrainAgeNeXt on lesion-filled images (Fig. 6A). When comparing the original brain age with the brain age obtained from lesion-filled images, we observed a strong correlation between the two values (Spearman’s ρ = 0.99). The mean difference between them was minimal (0.3 ± 1.3 years), and this difference was not significantly associated with lesion volume (r = 0.16,*p*= 0.08). Thus, we used the non-lesion-filled images to predict brain age for the subsequent analyses.

For the LCL cohort, we predicted brain age on both 3T and 7T MRI scans of the same individuals (Fig. 6B). The two predictions were strongly correlated (Spearman’s ρ = 0.99,*p*< 0.001) with a mean absolute difference of 1.5 ± 1.0 years (Fig. 6B).

**Fig. 7.**
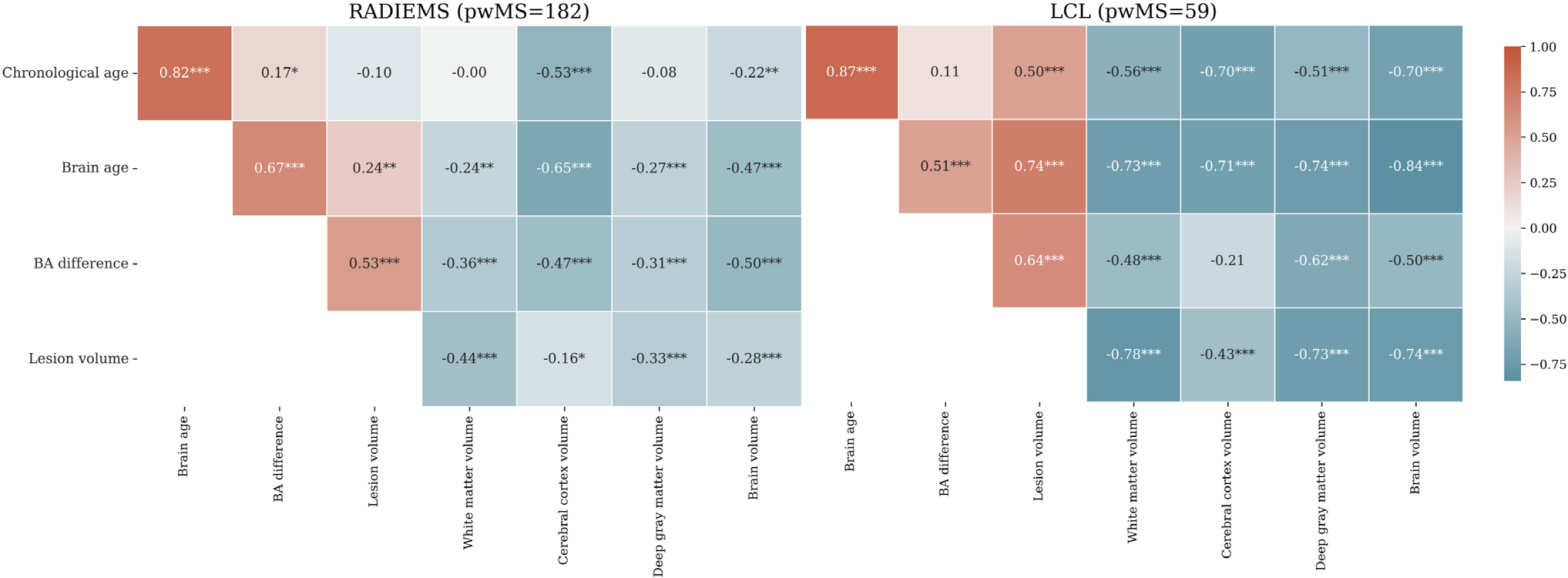
Brain age difference is correlated with brain volumetric measures in pwMS. Heatmap showing Spearman’s correlation coefficient between chronological age, brain age, brain age difference, and MRI markers for the RADIEMS (left) and LCL (right) cohorts. All volumetric measures are normalized to intracranial volume. Significance level: *p< 0.05, **p< 0.01, ***p< 0.001, all adjusted for multiple comparisons using the false discovery rate method.

We further investigated the correlations between chronological age, brain age, and BAD with MRI measures (Fig. 7). The Long 7T cohort was excluded from the subsequent analysis due to its small sample size (n = 28). In both the LCL and RADIEMS cohorts, chronological age, brain age, and BAD showed negative associations with normalized brain volume (NBV), deep gray matter volume, cortical volume, and white matter volume (Fig. 7). Importantly, brain age had stronger correlations with these volumetric measures compared to chronological age. Both brain age and BAD were strongly correlated with white matter lesion volume in both cohorts (*p*< 0.001). The strength of the correlations between chronological age and brain age with volumetric measures was generally stronger for the LCL cohort, while correlations with BAD were similar in the two cohorts.

We then examined the relationship between BAD and its interaction with disease duration on NBV and lesion volume using a generalized linear model. When combining the two cohorts and stratifying the subjects by disease duration (<6 years vs ≥6 years), we found an association between increased BAD and decreased NBV in both early MS (unstandardized B = -0.0023, 95% CI: -0.003 to -0.002,*p*< 0.001) and late MS groups (unstandardized B = -0.0021, 95% CI: -0.004 to -0.001,*p*= 0.007). In contrast, increased BAD showed a significant association with higher lesion volume in both groups. The association was moderate in the early MS group (unstandardized B = 784.13, 95% CI: 639.87 to 928.39,*p*< 0.001) and stronger in the late MS group (unstandardized B = 1051.37, 95% CI: 571.03 to 1531.72,*p*< 0.001).

**Fig. 8.**
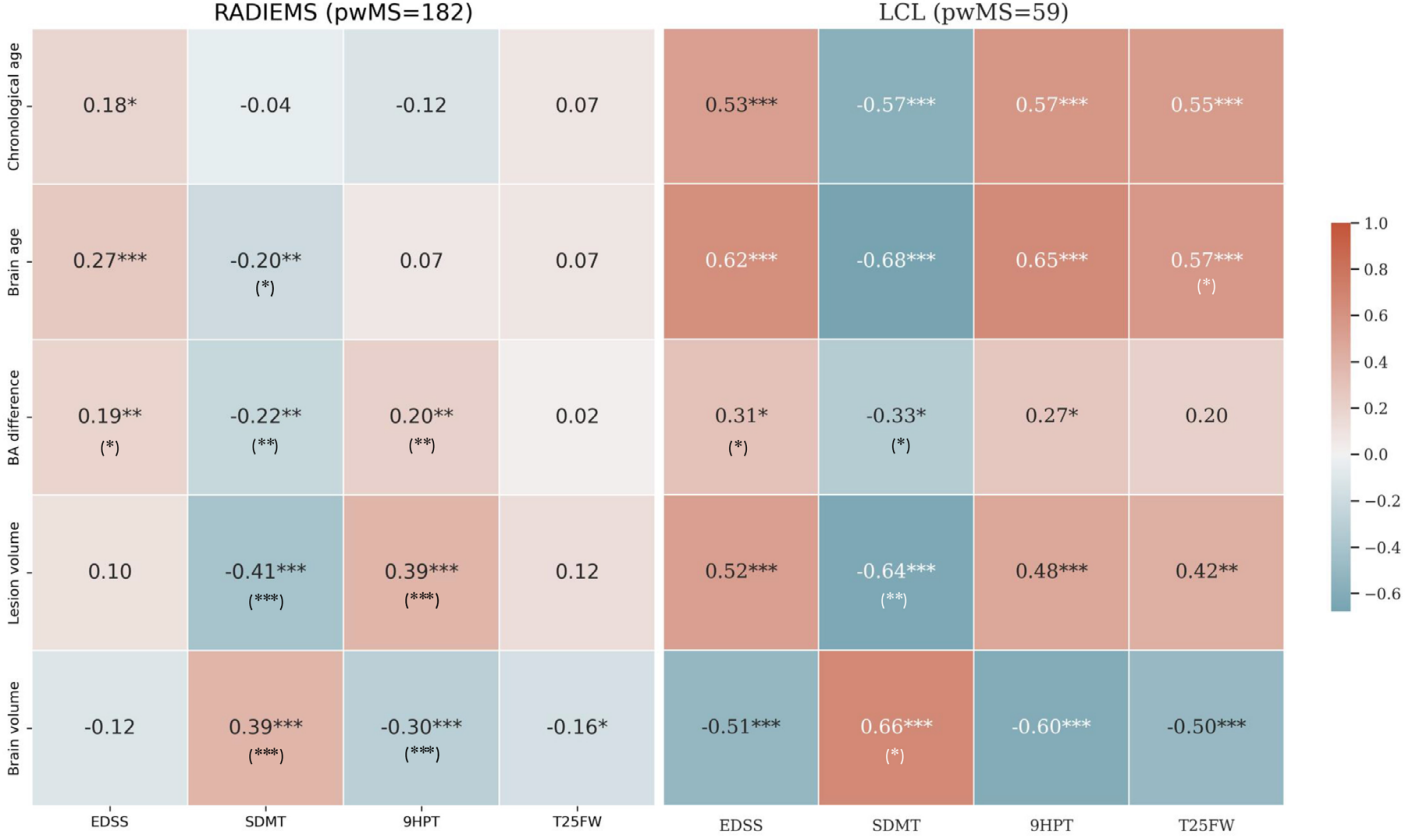
Brain age difference is associated with disability in pwMS. Heatmap showing Spearman’s correlation coefficient between chronological age, brain age (BA), brain age difference, and clinical measures for the LCL and RADIEMS cohorts. EDSS: Expanded Disability Status Scale; SDMT: Symbol Digit Modalities Test; 9HPT: Nine-Hole Peg Test; T25FW: Timed 25-Foot Walk. For EDSS, 9HPT, and T25FW, higher scores indicate worse performance whereas for SDMT, lower scores indicate worse performance. Significance level: *p< 0.05, **p< 0.01, ***p< 0.001, all adjusted for multiple comparisons using the false discovery rate method. Asterisks in parentheses indicate significance after adjusting for age.

Further, we analyzed relationships between brain age and four measures of disability: EDSS, SDMT, 9HPT, and T25FW (Fig. 8). The two MS cohorts exhibited similar trends, but the strength of the associations varied between them, with LCL showing stronger correlations. BAD was significantly correlated with SDMT, EDSS, and 9HPT in both cohorts.

#### 3.2.2 Longitudinal analysis

We analyzed the predicted brain age for all subjects with MS from RADIEMS and LCL with data available from at least two timepoints (*n*= 200, mean time interval between sessions = 2.85 years, range 1.43−5.51 years). To assess the variability in brain age longitudinal change across individual subjects, we computed the annual brain age change for each subject separately. The mean annual brain age change rate was 1.15 years (IQR 0.96, 1st quartile 0.68, 3rd quartile 1.64), with a 95% confidence interval of 1.05 to 1.26 years. This is statistically different from 1 (one-sample t-test,*p*= 0.003), revealing that in pwMS, brain aging is about 15% faster than chronological aging. The values for each MS cohort are reported inSupplementary Table 2. We visually inspected the outliers identified in this analysis and found that some exhibited artifacts or a noticeable bias field.

Thirty-eight subjects from the LCL cohort had both 3T and 7T scans at baseline and follow-up visits. The subject-wise mean difference between 3T and 7T annual brain age change was -0.1 years (Supplementary Figs. 2 and 3). No significant difference in annual brain age change was found between 3T and 7T MRI (*p*= 0.6).

**Fig. 9.**
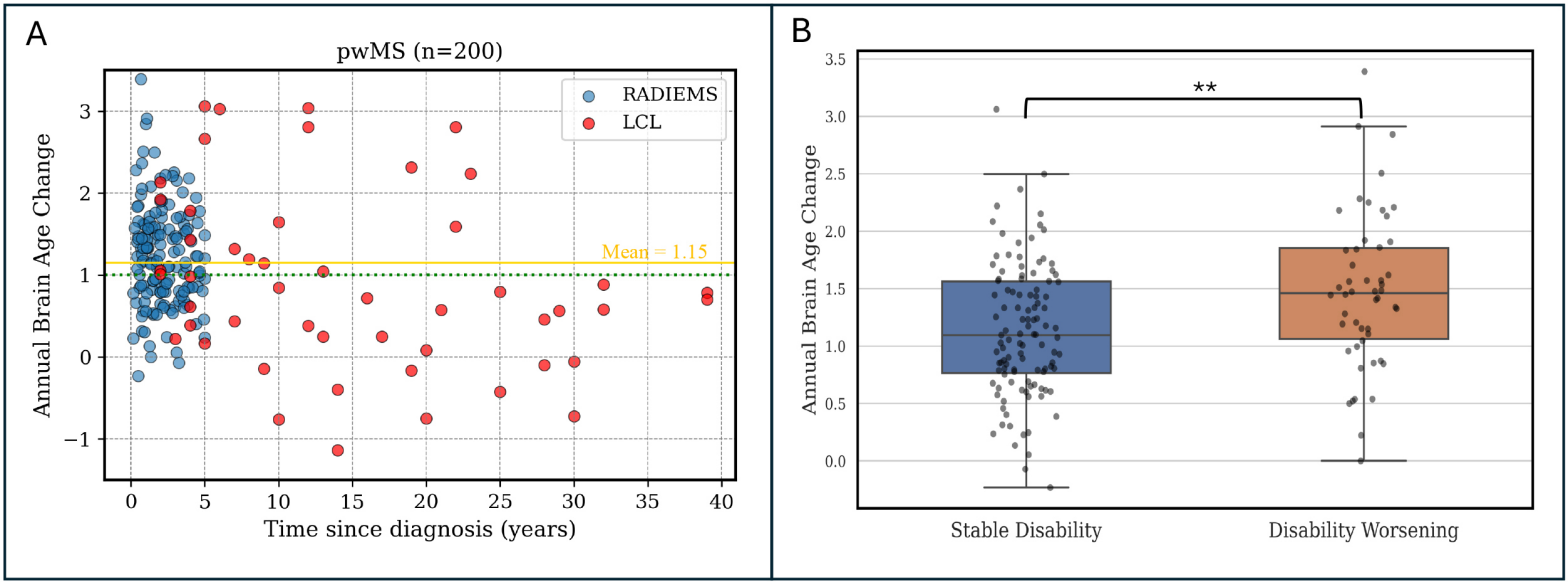
Brain aging is accelerated in MS and is associated with disability worsening. (A) Longitudinal brain age analysis reveals that individuals with MS (n = 200) have accelerated brain aging (mean annual brain age change = 1.15). The green dotted line shows the annual chronological age change, and the solid gold line shows the mean annual brain age change in pwMS. (B) Subjects with early MS (diagnosis <6 years,*n*= 163) are divided into two groups: those showing disability worsening, indicated by an increase in EDSS of ≥1.0 for baseline EDSS <6.0 or ≥0.5 for baseline EDSS ≥6.0, or 20% increase in T25FW, or 20% increase in 9HPT; and those whose clinical assessment remained stable. The subjects who experienced disability worsening between the two visits had a significantly greater annual brain age change (1.48) than those who had no disability worsening (1.14,*p*< 0.01). Significance level: **p< 0.01.

When dividing pwMS into early MS (disease duration of less than 6 years) and longstanding MS (disease duration greater than 6 years), we observed a statistically significant difference (*p*= 0.004) in the annual brain age change between these two groups (Fig. 9A). The early MS group had a mean annual brain age change of 1.24 ± 0.64 years, compared to 0.75 ± 1.08 years for the longstanding MS group.

Additionally, we categorized all pwMS based on disability progression (Goldman et al., 2019;Montalban et al., 2017). pwMS were classified as exhibiting disability worsening if there was an EDSS increase of ≥1.0 for baseline EDSS <6.0 or ≥0.5 for baseline EDSS ≥6.0, or a 20% increase in T25FW or 9HPT. In early MS, individuals with disability worsening had a greater brain age increase per year (1.48 ± 0.68 years) compared to individuals that remained stable (1.14 ± 0.60 years,*p*< 0.01) (Fig. 9B). No difference between the groups was observed in longstanding MS (*p*= 0.08,Supplementary Fig. 4).

## 4 Discussion

In this work, we propose a novel brain age estimation method, coined BrainAgeNeXt, inspired by the state-of-the-art convolutional neural network framework MedNeXt (Z. Liu et al., 2022;Roy et al., 2023). The proposed BrainAgeNeXt model outperformed other state-of-the-art deep learning approaches in predicting brain age across a large, diverse testing dataset of 1,523 HV MRI scans. Of note, two of the three brain age models used for comparison with BrainAgeNeXt were trained on different datasets. Although the testing set was unseen during training and the same for each model, differences in the training datasets may have introduced variability in the models’ performance that is unrelated to the capabilities of each approach. BrainAgeNeXt also achieves a similar MAE to other leading brain age models reported in recent reviews (Soumya Kumari & Sundarrajan, 2024;Tanveer et al., 2023), highlighting its competitive performance while uniquely leveraging 7T MRI data. This highlights the robustness and generalizability of BrainAgeNeXt, even when tested on MRI scans from various sites while preserving individuals’ differences. The findings of our study provide new insights into the application of deep learning-based models for brain age estimation, particularly in individuals with MS.

BrainAgeNeXt is a convolutional neural network inspired by the MedNeXt framework. While the original MedNeXt architecture was initially designed for segmentation tasks, its applicability to regression tasks such as brain age prediction is supported by its ability to effectively extract hierarchical features from 3D medical images and aggregate spatial information. The addition of global pooling and fully connected layers at the bottom of MedNeXt’s encoder transforms the spatially distributed features into a scalar output, making the model well-suited for a regression task such as brain age prediction. One of the key advantages of BrainAgeNeXt is its ability to learn directly from raw MRI data without relying on pre-extracted volumetric features. This approach mitigates the issues associated with brain parcellation variability and facilitates the model’s applicability to real-world clinical settings. Among the algorithms compared, brainageR utilizes parcellation features which are more susceptible to image artifacts and segmentation variability. The data augmentation techniques employed during BrainAgeNeXt training, including spatial transformations and the simulation of MRI artifacts, further contributed to the model’s robustness, enabling it to generalize well across different levels of image quality. BrainAgeNeXt is the best-performing approach even in the presence of motion artifacts, which considerably degrade the performance of voxel-based morphometry methods such as brainageR (Moqadam et al., 2024). However, BrainAgeNeXt’s performance is still significantly degraded when comparing artifact-free images to those with strong motion artifacts, highlighting the need to improve its robustness to severe artifacts. The worse performance of brainageR with motion-degraded images is likely due to the influence of motion on brain parcellation performed by SPM12 (Schmidt et al., 2012), which uses a Bayesian framework for tissue classification and anatomical parcellation. Motion artifacts can significantly degrade tissue contrast, making accurate segmentation challenging and brain age estimation less reliable. Additionally, our relatively smaller but diverse training dataset, which includes 1.5T, 3T, and 7T MRI scans from multiple sites, may have helped mitigate overfitting. In contrast, Pyment, while trained on over 34,000 scans, derived a large portion of its data from the UK Biobank. This likely introduced site- or scanner-specific biases due to the shared scanner and acquisition parameters across a significant subset of its training dataset.

Our study also highlights the importance of ultra-high field MRI in brain age modeling. To our knowledge, BrainAgeNeXt is the first deep learning-based approach trained with 7T data, resulting in improved performance on 7T brain age predictions compared to other state-of-the-art models. The 7T MRI testing dataset, however, is limited, and larger healthy volunteer datasets are required to validate the generalizability of these findings in the future. Additionally, datasets with paired 3T and 7T MRI scans from the same healthy subjects should be analyzed to further validate our findings. Of note, BrainAgeNeXt requires a fixed size input and thus it does not fully exploit the full 7T MRI resolution. CNN architectures with flexible input dimensions could better leverage the full spatial resolution of 7T MRI, potentially improving the accuracy of brain age predictions. Whether brain age predictions from 7T MRI are more sensitive to accelerated aging still remains to be explored. 7T MRI also allows the assessment of advanced MS biomarkers, such as cortical lesions, which are very difficult to quantify at lower magnetic field strengths (Beck et al., 2020). Thus, 7T MRI-derived brain age could be combined with other MS markers in the future to increase its prognostic value.

We found that brain age difference was correlated with volumes of several key brain structures. Specifically, brain age difference was positively correlated with CSF volume and negatively correlated with cerebral cortex and deep gray matter volumes. This aligns with the known patterns of the normal aging process, where increased CSF volume reflects brain atrophy, including shrinking of the cerebral cortex and loss of deep gray matter. These structural changes are indicative of age-related degeneration, suggesting that HV with higher brain age difference values exhibit more pronounced aging-related brain changes. Thus, the ability of BrainAgeNeXt to capture these associations highlights its potential utility in identifying deviations from typical healthy aging trajectories.

Recently, there has been growing interest in brain age in the context of MS (Brier et al., 2023;Cole et al., 2020;Denissen et al., 2021). Several studies have shown that brain age difference is greater in pwMS compared to HV. Brain age difference has also been associated with brain atrophy, physical disability, and cognitive performance, as measured by the SDMT, in pwMS (Denissen et al., 2021). In two independent longitudinal studies,Cole et al. (2020)andBrier et al. (2023)found that a higher brain age difference at baseline was associated with greater subsequent disability accumulation. This evidence suggests that brain age is a potentially useful metric to measure structural changes in the brain that are associated with disability in MS. To characterize brain age in MS, we tested BrainAgeNeXt on three longitudinal cohorts of subjects with MS, for a total of 273 individuals. The cross-sectional analysis revealed a significantly greater brain age difference in MS compared to healthy volunteers. Individuals with MS exhibited at baseline an average brain age approximately 4 years greater than their chronological age. Our results are consistent with previous studies (Brier et al., 2023;Cole et al., 2020;Denissen et al., 2021), emphasizing the accelerated brain aging process associated with MS. We also analyzed the impact of lesion-filled images on brain age predictions. Although white matter lesion volume was strongly correlated with brain age difference, we found that lesion-filling did not significantly change brain age predictions, similarly to what was observed in the literature (Cole et al., 2020). This suggests that while MS lesion burden is associated with accelerated aging, the lesions themselves are not the primary drivers of BrainAgeNeXt’s predictions.

Our analysis revealed that brain age is a stronger predictor of normalized brain volume than chronological age across the LCL and RADIEMS cohorts. This stronger correlation suggests that brain age provides a better representation of the neurobiological aging process than chronological age in pwMS. The finding highlights the added value of brain age over chronological age in assessing pwMS, as it reflects the underlying pathological processes more accurately. Additionally, both brain age and brain age difference are negatively associated with the normalized volume of key brain structures, including deep gray matter, cortex, and white matter. This consistent pattern of atrophy highlights the combined impact of aging and neurodegeneration in MS. Importantly, we observed significant associations between BAD, normalized brain volume, and lesion volume across different stages of MS. BAD was consistently associated with reduced normalized brain volume, reflecting the combined impact of neurodegeneration and accelerated brain aging. Additionally, BAD showed a stronger association with lesion volume in subjects with longstanding MS. This suggests that, in the later stages of the disease, when lesion burden is higher, lesions play a greater role in driving brain age. We hypothesize that this may be due to greater variability in lesion volume, which is not present early in the disease. Additionally, the damage caused by lesions may have had more time to result in downstream neurodegeneration, which could be reflected in BAD. These findings underscore the potential of BAD as a marker for both global atrophy and neurodegeneration, capturing the progressive and multifaceted nature of MS.

The longitudinal brain age analysis demonstrates that, on average, brain age tends to increase at a considerably faster rate than chronological age in early MS (<6 years since diagnosis), while this annual change may decrease in the later stages of the disease. However, as this longitudinal analysis compares cohorts from different institutions, variability may be introduced. Additionally, confounding factors, such as treatment type, could influence the observed trends, highlighting the need for larger cohorts to validate these results. Among all subjects with MS, the annual brain age change showed a wide IQR of 0.96, highlighting an inter-subject variability in brain aging rates, the drivers of which will be important to explore in the future. This provides additional evidence for the potential utility of brain age modeling in monitoring disease progression, particularly in its early stages. No significant differences were found when comparing the annual change in brain age predicted from 3T and 7T MRI scans. This confirms the robustness of BrainAgeNeXt across different magnetic field strengths.

Brain age difference is associated with several disability measures in both early and longstanding MS, suggesting that brain age may offer valuable prognostic information. The strongest correlations were found with SDMT, a measure of cognitive function, in line with previous studies (Cole et al., 2020;Denissen et al., 2021). Notably, brain age was more closely associated with all disability measures than chronological age. Additionally, in early MS, a greater annual brain age increase was linked to worsening disability. In the future, by identifying individuals with accelerated brain aging, clinicians may be able to implement early interventions and personalized treatment plans to mitigate the progression of cognitive impairment. Moreover, longitudinal brain age estimation could serve as an endpoint in clinical trials to assess the efficacy of therapeutic interventions and lifestyle modifications. Our analysis of the relationships between brain age and MRI and clinical measures was conducted using two datasets (RADIEMS and LCL) that differed in site as well as in study design, with RADIEMS including only participants within 5 years of MS diagnosis and LCL including mostly participants with longstanding disease. Thus, further studies are needed to determine whether the differences we observed between cohorts are due to effects of disease duration, other clinical factors, or variations in MRI acquisition.

Despite the promising results, several limitations should be acknowledged. The healthy volunteers and MS datasets used for training and validation predominantly consisted of European and American individuals, which may limit the generalizability of our findings to other ethnicities and populations. Future studies should aim to include a more diverse range of cohorts to ensure the broad applicability of brain age models. Additionally, while the BrainAgeNeXt ensemble demonstrated strong performance, there was a moderate variability between the predictions of the five individual models. Of note, the variability in sample size across different MRI sequences and field strengths, particularly the limited representation of 7T data, may have introduced biases that could limit the model’s ability to generalize across imaging modalities. Future work will investigate the causes of this variability, including potential model biases and dataset characteristics, to further enhance the stability and consistency of brain age predictions. While BrainAgeNeXt demonstrated excellent performance in predicting brain age, the clinical utility of brain age as a prognostic biomarker for MS requires further validation through longitudinal studies with larger cohorts and evaluation in the context of interventional clinical trials. Moreover, while we assessed correlations between brain age predictions and volumetric features for biological interpretation, finer-grained explainability methods, such as Gradient-weighted Class Activation Mapping (Grad-CAMs), could further enhance our understanding of the focus of BrainAgeNeXt and its biological implications. Finally, the performance of BrainAgeNeXt should be evaluated in future studies using clinical-grade scans and including other T1w sequences.

In conclusion, BrainAgeNeXt, which we have made publicly available (https://github.com/FrancescoLR/BrainAgeNeXt), represents a significant advance in brain age modeling. It offers a robust and accurate method for estimating brain age from diverse MRI data, including 7T MRI. Its application to individuals with MS demonstrates the potential for brain age as a valuable biomarker for assessing accelerated aging and MS disability progression. By requiring only a T1-weighted MRI to compute brain age, we hope to advance brain age research further and facilitate its application to neurological conditions.

## Supplementary Material

Supplementary Material

## Data Availability

BrainAgeNext’s code and its trained model are available at the following GitHub repository:https://github.com/FrancescoLR/BrainAgeNeXt. Most datasets used are publicly available. The private datasets are available upon reasonable request and require appropriate ethical approval or data use agreements.

## Footnotes

1
https://github.com/FrancescoLR/BrainAgeNeXt
