# Supplementary material for: BrainAgeNeXt: Advancing brain age modeling for individuals with multiple sclerosis
Source: Imaging Neurosci (Camb). 2025 Feb 25;3:imag_a_00487. doi: 10.1162/imag_a_00487 (PMC12319810; doi:10.1162/imag_a_00487)
Supplement: Supplementary Material [file imag_a_00487-supp.pdf]

Supplementary material

	Testing dataset (*n* = 352)		
Model	MAE (years)	BA vs CA (Pearson's *r*)	BAD vs CA (Pearson's *r*)
BrainAgeNeXt	**3.05**	**0.982*** **	**-0.018**
DenseNet	3.61	0.973*	-0.252
pyment	3.45	0.978***	-0.209
brainageR	4.46	0.965	-0.202

Supplementary Table 1. Performance of BrainAgeNeXt vs state-of-the-art models. This smaller testing dataset includes all subjects not present in brainageR training dataset and who are 18 years old or older. Bold font indicates the best performance for each metric across models. MAE: mean age error. BA: brain age. CA: chronological age. BAD: brain age difference. Significance levels: * p < 0.05, ** p < 0.01, *** p < 0.001.

MS Cohort	Time since diagnosis (years)	Time between MRI sessions (years)	BA Change per year (years)
RADIEMS	2.23 ± 1.46	3.21 ± 0.40	1.23 ± 0.62
LCL	14.22 ± 10.86	2.18 ± 0.51	0.90 ± 1.06

Supplementary Table 2. Brain age increases more rapidly at the early stages of multiple sclerosis.

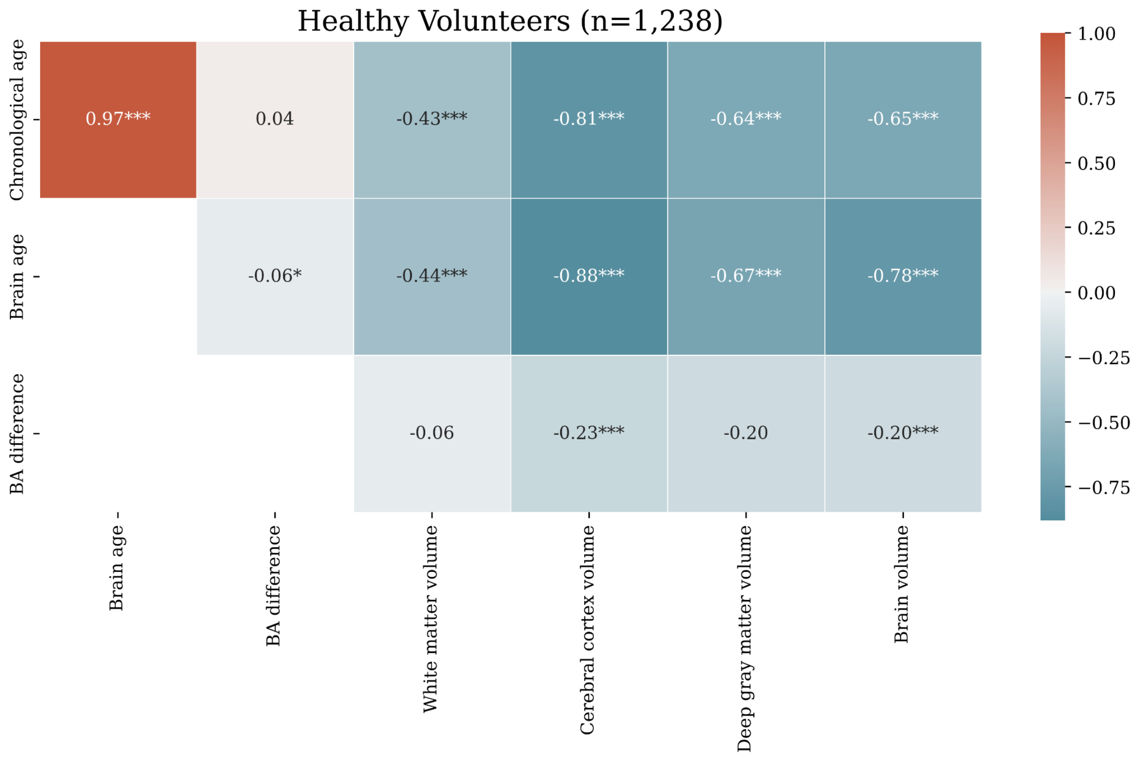

Supplementary Figure 1. Brain age difference is correlated with cerebral cortex and brain volume in healthy volunteers. Heatmap showing Spearman's correlation coefficient between chronological age, brain age, brain age difference, and brain volumetric features for the healthy volunteers aged 21 and older. Significance level: * p < 0.05, ** p < 0.01, *** p < 0.001, all adjusted for multiple comparisons using the false discovery rate method.

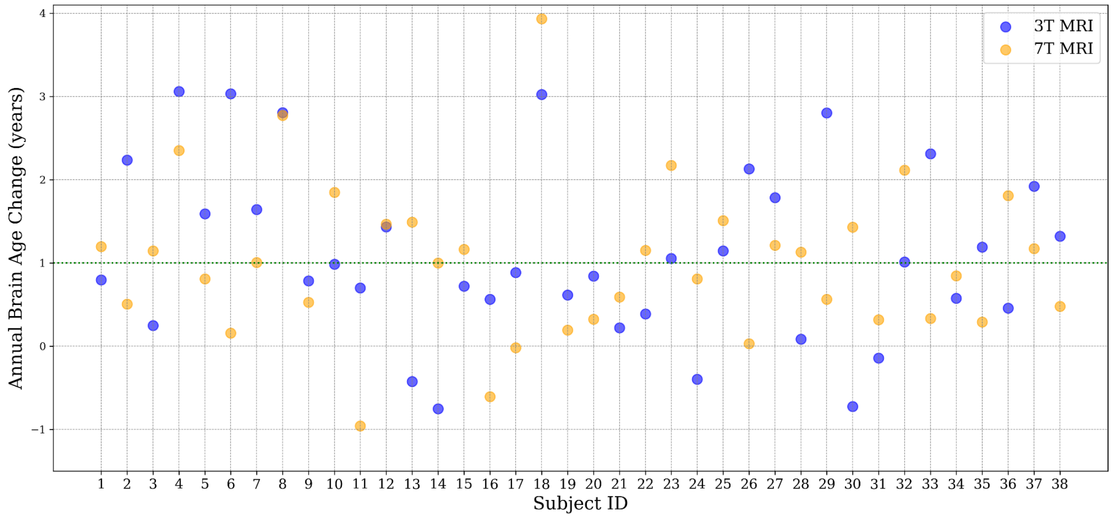

Supplementary Figure 2. The annual change in brain age does not significantly differ when comparing 3T and 7T MRI brain age (*p*=0.6). The plot shows a comparison of 38 pwMS from the LCL cohort imaged at both 3T and 7T at two timepoints.

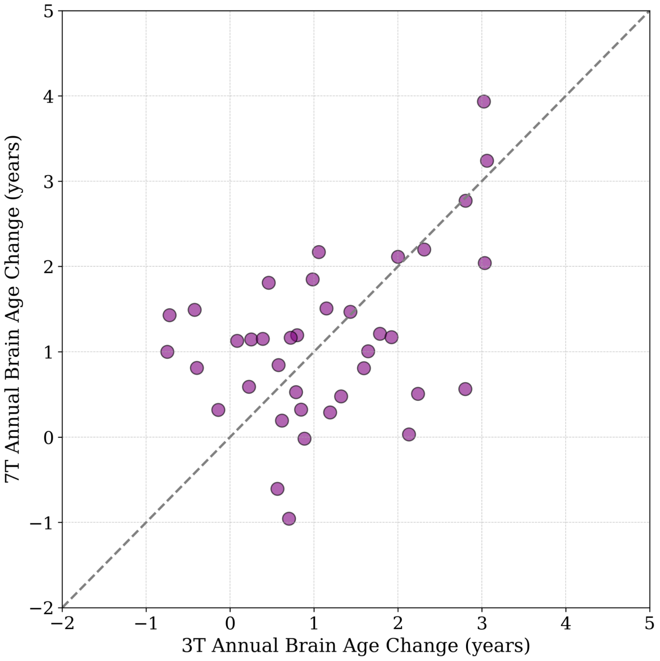

Supplementary Figure 3. Scatter plot showing the annual brain age change computed from 3T and 7T MRI scans of people with MS (*n* = 38).

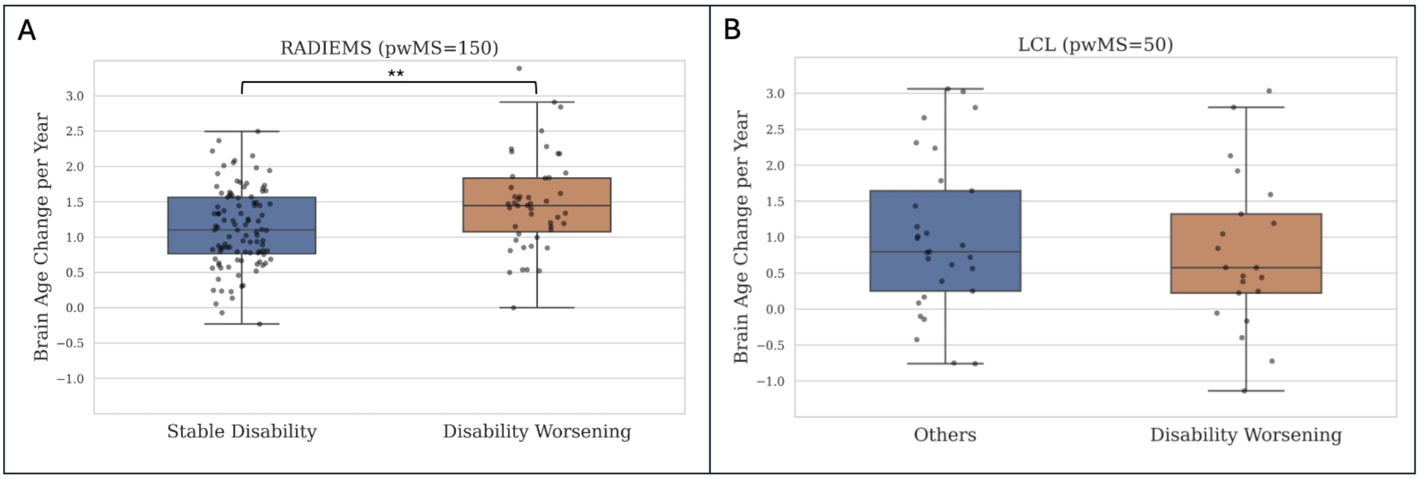

Supplementary Figure 4. The annual brain age change is greater for subjects with disability worsening compared to stable ones in RADIEMS, whereas no differences are observed in LCL. Significance level: ** $p < 0.01$.
